# Reduction in Opioid Prescribing After Removal of Electronic Health Record Defaults Among Emergency Department Residents, Advanced Practice Providers, and Attendings

**DOI:** 10.7759/cureus.109706

**Published:** 2026-05-26

**Authors:** Kito Lord, James R Walker, Michael Reich, Justin Griner, Chantay Smartt, Mark F Brady

**Affiliations:** 1 Emergency Medicine, The University of Tennessee Health Science Center, Memphis, USA; 2 Pharmacy, Regional One Health, Memphis, USA; 3 Emergency Medicine, The Warren Alpert Medical School of Brown University, Providence, USA

**Keywords:** electronic health record, opioids, overprescribing, prescribing behavior, quality improvement

## Abstract

Introduction

Opioid prescription reduction is a high-priority quality improvement area in the emergency department (ED) with significant opportunities for harm reduction. While electronic health records (EHR) default dispense quantities have been used to help practitioners become more efficient, automated prescription quantities may influence prescribers to provide more than what they would otherwise deem necessary. We sought to evaluate the prescribing behavior of residents, advanced practice providers (APPs), and attending physicians after the removal of the default dispense quantity of the most prescribed opioid in the ED.

Methods

This is a single-center retrospective pre-post observational study in the ED of the four months before and the four months after removing the default dispense quantity of 20 tablets of oxycodone/acetaminophen per prescription. We compared the changes in prescribing patterns of emergency medicine (EM) attendings, EM APPs, EM residents, surgical attendings, surgical residents, and surgical APPs.

Results

During the study period, there were a total of 2,468 prescriptions for oxycodone/acetaminophen, resulting in 41,612 tablets prescribed. There were 1,314 prescriptions for oxycodone/acetaminophen before and 1,154 prescriptions written after the removal of the default dispense quantity. The average number of tablets per prescription was reduced from 18.16 to 15.35 after the removal of the default dispense quantity. There was no significant difference in the number of prescriptions written before and after the intervention in terms of patient age, gender, race, Emergency Severity Index (ESI) level, or department of the prescriber. An overall 15% reduction in the number of tablets of oxycodone/acetaminophen prescribed was observed. This reduction was significant among EM attendings, EM residents, EM APPs, and surgical attendings. There was no significant change among surgical residents and surgical APPs.

Conclusion

Removing the default dispense quantity was associated with a reduction in the amount of oxycodone/acetaminophen prescribed in most but not all categories of prescribers. While lower default quantities have reduced opioid prescription quantities, a default dispense quantity of 20 tablets may lead to overprescribing. As practice patterns and recommended guidelines change, default dispense quantities should periodically be evaluated for unintended influence on residents, APPs, and attendings' behavior.

## Introduction

The opioid epidemic continues to be a leading cause of preventable deaths in the United States [1]. With more than 100,306 opioid overdose deaths and a 28.5% increase from the previous 12-month period in 2020, the opioid crisis continues to be a significant source of morbidity and mortality [2]. The emergency department (ED) plays a vital role in coordinated efforts to prevent and treat opioid dependence [3-6]. Opioid-naive patients prescribed an opioid at ED discharge are at increased risk of using prescription opioids one year later [3]. Studies have also demonstrated a link between abuse of prescribed opioids and heroin use [5,7,8]. Despite a decrease in opioid prescriptions from the ED, more than 11 million individuals admit to misusing an opioid prescription [4,9]. 

There have been several national and statewide initiatives aimed at reducing opioid use that may be contributing to the decline of opioid prescribing on a federal, state, and local level [10]. While the Centers for Disease Control and Prevention (CDC) and other bodies have suggested guidelines for chronic pain, quantities of prescribed opioids still vary by state, facility, and diagnosis [9-11]. Additionally, prescribers at the same hospital may prescribe varying amounts of opioids for the same condition [12,13]. The electronic medical record (EMR) has been used to help reduce prescriber variation by providing suggested quantities of opioids. Electronic prescriptions using autopopulation or default dispense quantities can influence prescriber patterns [14-21]. 

While lowering the default tablet dispense quantity has been associated with lower quantities of opioids prescribed, there is no consensus regarding the optimal default quantity [14-18,20,22]. Too large of a default quantity could drive prescribers to provide higher quantities of opioids than are necessary. The impact of removing default dispense quantities has led to mixed overall results [21,23-25]. The impact of instituting, changing, or removing default dispense quantities may vary by training level and role. A default prescription quantity of 20 tablets of oxycodone/acetaminophen was initially created to help expedite prescriptions. We sought to explore ED opioid prescription behavior based on prescriber department and role following the removal of the default dispense quantity in the EMR.

## Materials and methods

Study design and setting 

This was a retrospective before-and-after study following a quality improvement intervention aimed at opioid reduction in an urban, academic tertiary referral center in Memphis, TN. The hospital is a Level I Trauma Center with an average volume of >50,000 visits per year. The department is staffed by emergency medicine (EM) residents, EM advanced practice providers (APPs), EM attendings and surgical attendings, residents, and APPs. During this study period, trauma-related injuries were staffed primarily by the trauma residents, attendings, and APPs. Institutional Review Board approval was obtained. 

Study population 

We included all patients arriving and discharged from the ED between the dates of November 1, 2017, and June 30, 2018, who received a prescription for the most frequently prescribed opioid: oxycodone/acetaminophen. Patients who were admitted to the hospital, transferred to another facility, or expired were excluded. Patients with incomplete records or when the prescriber could not be identified were excluded. The default dispense quantity for oxycodone/acetaminophen for 20 tablets was removed on March 1, 2018, requiring physicians and APPs to self-select the quantity of tablets prescribed for their patients in the emergency department. 

The primary outcome was the number of oxycodone/acetaminophen tablets prescribed per prescription after the removal of the default dispense quantity on ED residents, APPs, and attendings. Descriptive patient characteristics, such as age, sex, and Emergency Severity Index (ESI) acuity, were collected. 

Statistical analysis 

Patient characteristics were summarized as frequencies and percentages for categorical variables and as mean (standard deviation (SD)) or median (interquartile range (IQR)) for continuous variables. Categorical variables were compared using the Chi-square test across the full distribution of each variable, including unknown categories where present. Continuous variables were compared using the Wilcoxon rank-sum test due to non-normal distribution.

The primary outcome was the quantity of oxycodone/acetaminophen tablets prescribed per prescription before and after removal of the default dispense quantity. Unadjusted differences in tablets prescribed per prescription were assessed using the Wilcoxon rank-sum test. Adjusted regression models were used to estimate the association between removal of the default dispense quantity and tablets prescribed per prescription after accounting for gender, race, prescriber position, and prescribing department. Multilevel linear regression was performed to evaluate differences in tablet reduction by prescriber role. A p-value ≤ 0.05 was considered statistically significant. The analysis was conducted in R version 3.5.3 (R Foundation for Statistical Computing, Vienna, Austria).

## Results

There were a total of 31,667 ED visits during the study period. Demographic characteristics of the overall ED population during the study period were as follows: the average age was 33.35 years (SD: 13.52), with 53.2% of patients being male. African Americans were the most frequently represented group (77.3%), followed by Caucasian (21.5%), with American Indian and Asian patients each representing 0.4%. The majority of patients were ESI level 3 (63.1%), followed by ESI level 4 (25.4%), ESI level 2 (7.2%), ESI level 5 (4.0%), and ESI level 1 (0.4%). These values reflect the overall ED population and differ from the prescription-level characteristics presented in Table 1, which are limited to patients receiving oxycodone/acetaminophen prescriptions. Of the 31,667 ED visits, approximately 12% (3,800) resulted in discharge with an opioid prescription. Oxycodone/acetaminophen was the most frequently prescribed opioid, representing 65% (2,468) of all opioid prescriptions.

**Table 1:** Oxycodone/acetaminophen patient characteristics before and after default dispense quantity removal IQR: interquartile range; ESI: Emergency Severity Index Counts may vary by variable due to missing demographic or clinical data. Prescription quantity and prescriber characteristics are presented at the prescription level

		Default dispense quantity present	Default dispense quantity removed	Statistical test	p-value
Age (median (IQR))		24.00 (19.00, 34.00)	24.00 (19.00, 35.00)	Wilcoxon rank-sum	0.619
Age (mean (SD))		28.25 (12.52)	28.60 (12.66)	Wilcoxon rank-sum	0.568
Gender (n (%))	Female	459 (50.1)	412 (50.4)	Chi-square	0.953
	Male	457 (49.9)	406 (49.6)		
Race (n (%))	African American	675 (80.3)	593 (81.6)	Chi-square	0.793
	American Indian	5 (0.6)	3 (0.4)		
	Asian	3 (0.4)	4 (0.6)		
	White	158 (18.8)	127 (17.5)		
ESI (n (%))	ESI 1 resuscitation	1 (0.1)	3 (0.4)	Chi-square	0.264
	ESI 2 emergent	50 (5.5)	56 (6.8)		
	ESI 3 urgent	692 (75.5)	594 (72.6)		
	ESI 4 semi-urgent	136 (14.8)	139 (17.0)		
	ESI 5 non-urgent	37 (4.0)	26 (3.2)		
Prescriber position (n (%))	Attending	418 (31.8)	337 (29.2)	Chi-square	<0.001
	Nurse Practitioner	236 (18.0)	259 ( 22.4)		
	Physician assistant	80 (6.1)	34 (2.9)		
	Resident	526 (40.0)	483 (41.9)		
Prescriber department (n (%))	Surgical department	738 ( 56.2)	676 ( 58.6)	Chi-square	0.242
	Emergency department	576 ( 43.8)	478 ( 41.4)		
Quantity (median (IQR))		20.00 (12.00, 20.00)	12.00 (10.00, 20.00)	Wilcoxon rank-sum	<0.001
Quantity (mean (SD))		18.16 (6.41)	15.35 (7.51)	Wilcoxon rank-sum	<0.001

There were 1,314 prescriptions for oxycodone/acetaminophen before the removal of the default dispense quantity and 1,154 prescriptions written after the removal of the default dispense quantity. Most prescriptions were written by surgical residents, followed by EM attendings. There was no significant difference in the number of prescriptions written by patient age, gender, race, ESI level, or prescriber department, though provider position was significantly associated with the number of prescriptions written.

Our primary outcome was the quantity of oxycodone/acetaminophen tablets prescribed before and after the removal of the EMR default. In the unadjusted analysis, there was a 15% decrease in the number of oxycodone/acetaminophen tablets prescribed per prescription, with a mean of 18.16 before the intervention vs. 15.35 after the intervention, leading to a decrease of 2.81 tablets per prescription (p < 0.001). In the adjusted regression model, removal of the default dispense quantity was associated with an estimated reduction of 3.28 per prescription after accounting for covariates of gender, race, prescriber position, and prescribing department. Multilevel linear regression at the prescriber level demonstrated a significant decrease in the quantity of tablets prescribed by EM attendings (-4.87 CI: -6.52 to -3.23), EM residents (-5.11 CI:-7.03 to -3.18), EM APPs (-6.25 CI: -8.02 to -4.49), and surgical attendings (-5.34 CI: -7.36 to -3.33). There were no statistically significant reductions in surgical residents and surgical APPs (Table 2).

**Table 2:** Multilevel linear Regression: oxycodone/acetaminophen APP: advanced practice provider

Role	Change in tablets prescribed per prescription	Confidence interval	p-value
Pre-/post-intervention	-3.28	(-3.93, -2.64)	<0.001
Emergency medicine attending	-4.87	(-6.52, -3.23)	<0.001
Surgical attending	-5.34	(-7.36, -3.33)	<0.001
Emergency medicine APP	-6.25	(-8.02, -4.49)	<0.001
Surgical APP	-0.17	(-1.91, 1.56)	0.844
Emergency medicine resident	-5.11	(-7.03, -3.18)	<0.001
Surgical resident	-0.27	(-1.88 , 1.33)	0.739

## Discussion

Our study found that when opioid prescribers were required to actively choose an appropriate quantity of opioids, there was a 15% overall reduction in oxycodone/acetaminophen tablets prescribed per prescription. These findings were most pronounced in the EM APPs, followed by surgical attendings, EM attendings, and EM residents, respectively, suggesting they were most sensitive to the change in the EMR. A default dispense quantity of 20 tablets may have led each of these prescriber groups to prescribe more than they would have without EMR prompting. Although the reduction in tablets prescribed may seem modest at the individual level, these reductions at the population level translate to approximately 3,200 fewer opioid tablets prescribed within four months post-intervention. Even small decreases in opioid exposures across large patient populations may reduce excess unused tablets available for diversion or misuse. 

Despite an overall reduction in the quantity of oxycodone/acetaminophen prescribed, surgical APPs and surgical residents did not show a significant reduction in the prescribed quantity of oxycodone/acetaminophen. It could be that surgical residents and APPs may have had their prescribing patterns influenced by the previous default dispense quantity, or the quantity of 20 tablets was appropriately suited to their patient population. This may suggest the former rather than the latter, as there was a statistically significant reduction in prescriptions written by surgical attendings who would be treating similar populations. Additionally, other studies have found a similar difference between EM and non-EM trainees [26,27]. Our results suggest that the influence of EMR default dispense quantities varies by role and training level. This is important because residents and APPs prescribed the majority of opioids in the study. Healthcare leaders should consider the impact of training and role when determining EMR default dispense quantities. 

While the optimal default dispense quantity has not been established, our study suggests that a default of 20 tablets may unintentionally increase the quantity of opioids prescribed to patients discharged from the ED. Prescribers reduced their preference for the 20 tablet count by 65%, further suggesting EMR default dispense was driving prescriber behavior. Our study builds upon previous work, which demonstrated that the elimination of a default dispense quantity of 20 tablets led to decreased opioids prescribed [21]. It is likely that an EMR default dispense quantity of 20 tablets is larger than what most prescribers would give without a suggested amount. Two other studies demonstrated minimal impact of the removal of defaults [23,24]. This is likely due to a lower default dispense quantity of 15 and heightened awareness surrounding opioid prescription quantity following national guidelines [11]. Our work suggests that default dispense quantities should periodically be evaluated to ensure they are not driving attendings, residents, and APPs to overprescribe. 

Limitations 

There were a number of limitations to the study that may limit the generalizability. This was a retrospective, single-center study at a Level I trauma center with variable EM resident coverage. The study dates were limited due to legislative changes surrounding opioid prescriptions from the ED. While oxycodone/acetaminophen represented more than 63% of all opioids prescribed in the sample, it is possible that the trend in reduction of the number of tablets was not present across the other opioids. Additionally, the acuity and distribution of patient type may have varied by role and training. Feedback and communication varied based on role and may have influenced prescribing behavior. Potential confounders such as patient pain score, presenting diagnosis, injury severity, and emergency department crowding were not directly assessed and may have influenced the opioid quantities prescribed. Additionally, the study period coincided with an increasing national and state attention toward opioid stewardship. Secular trends in opioid prescribing may have contributed to reductions independent of the EHR intervention. 

## Conclusions

Removing the default dispense quantity of 20 tablets was associated with a reduction of oxycodone/acetaminophen quantities prescribed. The reduction varied between APPs, residents, and attendings. These findings suggest the default dispense quantity may have an unintended consequence of leading some physicians and APPs to prescribe higher quantities of opioids than necessary. Information technologists, pharmacists, healthcare administrators, and physician leaders should work together to frequently review the impact of default dispense quantities and tailor interventions for opioid prescribing reduction by the training level of the prescriber.
